# Effect of diacylglycerol acyltransferase 2 overexpression in 3T3-L1 is associated to an increase in mono-unsaturated fatty acid accumulation

**DOI:** 10.1186/2049-1891-5-29

**Published:** 2014-05-28

**Authors:** Zhiqi Zhang, Gang Shu, Xiaotong Zhu, Junming Guo, Han Cai, Songbo Wang, Lina Wang, Ping Gao, Qianyun Xi, Yongliang Zhang, Li Yuan, Qingyan Jiang

**Affiliations:** 1 ALLTECH-SCAU Animal Nutrition Control Research Alliance, College of Animal Science, South China Agricultural University, Guangzhou 510642, China; 2 College of life science, Xiamen University, Fujian province, Xiamen 361005, China

**Keywords:** DGAT2, Fatty acid composition, Overexpressing, Pig

## Abstract

**Background:**

Fatty acid (FA) composition is the most important parameter affecting the flavor and nutritional value of the meat. The final and the only committed step in the biosynthesis of triglycerides is catalyzed by diacylglycerol acyltransferase 2 (DGAT2). The role of DGAT2 in lipid accumulation has been demonstrated in adipocytes, However, little is known about the effect of DGAT2 on the FA composition of these cells.

**Methods:**

To investigate the role of DGAT2 in regulating lipid accumulation, FA composition and the expression of adipogenic genes, we cloned the open reading frame of the porcine *DGAT2* gene and established 3T3-L1 cells that overexpressed DGAT2. Cells were then cultured in differentiation medium (DM) without FA, with a mixture of FAs (FA-DM), or containing a ^13^C stable isotope-labeled FA mixture (IFA-DM). The FA composition of adipocytes was analyzed by gas chromatography–mass spectrometry and gas chromatography-isotope ratio mass spectrometry. Quantitative PCR and western blotting were employed to detect expression of adipogenic genes in 3T3-L1 adipocytes cultured with FA-DM for 12 d.

**Results:**

The triacylglyceride (TAG) content was significantly higher in 3T3-L1 adipocytes overexpressing DGAT2 than in control cells. When cultured in DM or FA-DM for 12 d, cells overexpressing DGAT2 showed a higher proportion of unsaturated FAs (C16:1 and C18:1). However, when cells overexpressing DGAT2 were cultured with FA-DM for 30 min, the FA composition was almost identical to that of controls. Further, the proportion of stable isotope-labeled FAs were similar in 3T3-L1 adipocytes overexpressing DGAT2 and control cells cultured in IFA-DM for 12 d. These results collectively indicate that the higher proportion of mono-unsaturated FAs, C16:1 and C18:1, may originate from de novo FA synthesis but not from the uptake of specific FAs from the medium. This hypothesis is further supported by evidence that both mRNA and protein expression of genes involved in FA synthesis (ACACA, FASN, SCD1, and A-FABP) were significantly higher in cells overexpressing DGAT2 than in control cells.

**Conclusions:**

In conclusion, our study revealed that TAG accumulation, the proportion of MUFAs, and the expression of adipogenic genes were higher in 3T3-L1 cells overexpressing DGAT2 than in control cells.

## Introduction

Meat quality depends on various sensory and chemical parameters, including color, tenderness, and the content of flavoring substances [1]. Fatty acid (FA) composition is one of the most important parameters affecting meat quality. The proportion of saturated, monounsaturated, and polyunsaturated FAs in the diet is reported to have important effects on human health. For instance, high intake of saturated FAs can elevate plasma cholesterol, which can have harmful cardiovascular effects [2]. Further, foods rich in monounsaturated FAs (MUFAs) may decrease platelet aggregation [3], increase bleeding time [4], and increase fibrinolysis [5]; thereby preventing thrombogenesis.

The FA composition of cells is determined by phospholipid metabolism [6,7], FA synthesis [8], and FA transport [9-13]. Diacylglycerol acyltransferase (DGAT1 and DGAT2) catalyzes the final step in triacylglyceride (TAG) formation through the acylation of diacylglycerol (DAG) [14,15]. DGAT1 plays an important role in incorporating oleoyl-CoA into TAG [15,16]. In COS-7 cells, DGAT2 overexpression has been reported to significantly increase lipid accumulation [17]. In contrast, lipid storage in 3T3-L1 adipocytes was markedly decreased by DGAT2 knockdown [18]. The effect of DGAT2 overexpression on the FA composition of cells is unknown.

This study investigated the effect of DGAT2 overexpression on the FA composition of 3T3-L1 preadipocytes. mRNA and protein expression of adipogenic genes in cells overexpressing DGAT2 was also investigated. Our results revealed a crucial role of DGAT2 in the regulation of FA composition and adipogenic gene expression.

## Methods

### Generation of 3T3-L1 preadipocytes overexpressing DGAT2

The open reading frame region of DGAT2 was subcloned into pcDNA3.1(+) to produce pcDNA3.1(+)-DGAT2, which was then linearized and transfected into 3T3-L1 cells (CL-173, ATCC, USA). Cells transfected with pcDNA3.1(+)-DGAT2 and pcDNA3.1(+) were treated with G418 ( 350 μg/mL) for 14 d until all non-transfected cells died. The selected transfected cells were then cultured in growth medium or differentiation medium supplemented with G418 (150 μg/mL) for further analysis.

### Cell culture

3T3-L1 preadipocytes were cultured and induced to differentiate as previously described [19]. The cells were cultured in Dulbecco’s modified Eagle’s medium (DMEM)/F12 containing 10% fetal bovine serum (FBS, Life Technologies, Grand Island, NY, USA). After reaching confluence, the cells were induced to differentiate by incubation in DMEM/F12 supplemented with 10% FBS, 0.174 μmol/L insulin, 0.5 mmol/L methylisobutylxanthine, and 1 μmol/L dexamethasone (Sigma- Aldrich, St. Louis, MO, USA). Two days after the initiation of differentiation, dexamethasone and methylisobutylxanthine were withdrawn from the medium. The differentiated 3T3-L1 cells were incubated with three differential medium until the cells matured (12 d): DMEM/F12, 10% charcoal-stripped FBS, and 0.174 μmol/L insulin (DM) (Life Technologies, Grand Island, NY, USA), DM supplemented with unlabeled FA (FA-DM), or DM supplemented with one of the stable isotope-labeled FA mixtures (FAM-DM). FA-DM contained 10 μmol/L C_16_-palmic acid, C_16_-palmitoleic acid, C_18_-stearic acid, C_18_-oleic acid, C_18_-linoleic acid, and C_20_-arachidic acid (Sigma-Aldrich, St. Louis, MO, USA). IFA-DM contained 10 μmol/L ^13^C_16_-palmic acid, ^13^C_18_-stearic acid, ^13^C_18_-oleic acid, (Cambridge Isotope Laboratories, Tewksbury, MA, USA) C_16_-palmitoleic acid, C_18_-linoleic acid, and C_20_-arachidic acid. All FA mixtures were pre-complexed with 60 μmol/L FA-free bovine serum albumin (BSA, Sigma-Aldrich, St. Louis, MO, USA).

### Triglyceride analysis and Oil Red O staining

The cells were washed twice with Ca^2+^- and Mg^2+^-free PBS and lysed using 150 μL of RIPA lysis buffer (50 mmol/L Tris, 150 mmol/L NaCl, 1% Triton X-100, 1% sodium deoxycholate, 0.1% SDS, 1 mmol/L EDTA, and 1 mmol/L phenylmethylsulfonyl fluoride). The TAG and total protein content of the cells in each well were determined from cell lysates using commercial kits (Biosino Bio-Technology and Science Inc., Beijing, China) on a microplate reader (Thermo Labsystems MK3, Thermo Fisher Scientific Inc., Waltham, MA, USA) according to the manufacturer’s protocol. The TAG content of cells in each well was then normalized to the total protein. Mature adipocytes were rinsed twice with Ca^2+^- and Mg^2+^-free PBS and then fixed in 4% paraformaldehyde in PBS (w/v) for 30 min at room temperature to facilitate Oil Red O staining. A stock solution of 0.35% Oil Red O (Amresco, Inc., Solon, OH, USA) in isopropanol (w/v) was diluted in water (6:4, v/v) and added to the fixed cells for 1 h at room temperature. The cells were then washed with water and photographed.

### Analysis of cellular FA composition

Cellular lipids were extracted according to a previously described procedure [20], converted to FA methyl esters using BF_3_ and methanolic potassium hydroxide [21], and analyzed using gas chromatography (GC; model MSD-6890; Agilent, USA) equipped with an automatic injector. Aliquots of 1 μL were injected into the capillary column (30 m × 0.32 mm × 0.25 μm; DB-5 MS; Agilent) with cyanopropyl methyl silicone as the stationary phase. The column oven temperature was programmed to hold at 130°C for 1 min, increase from 130°C to 200°C at 5°C/min, and then hold at 200°C for 5 min. Helium was used as the carrier gas at a flow rate of 1 mL/min. The proportions of individual FAs were determined by measuring the peak area using ChemStation software.

### Stable isotope-labeled FA profile

Cellular lipids were extracted and converted to FA methyl esters, which were resolved by GC using a chromatograph (Model 6890, Agilent, USA) equipped with a 30 m × 0.32 mm × 0.25 μm DB-5 MS capillary column (Agilent, USA) and burned to generate CO_2_ and to detect the molecular ions of masses 44, 45, and 46 using isotope ratio mass spectrometry (IR-MS) (GV Instruments, UK). A mixed nominal sample (C_14_-myristic acid, C_30_-FA, C_21_-diolefine, C_26_-diolefine, and C_36_-diolefine; University of Illinois, USA) was used with a standardized isotope value of CO_2_ in the cylinder. The carbon isotope value of each FA was calculated using the formula δ = [(R_s_/R_R_) − 1] × 1,000, where δ and R_s/_R_R_ represent the carbon isotope and ^13^C/^12^C values, respectively, of an international nominal sample (Pee Dee Belemnite, South Carolina, USA). The data are relative to control cells transfected with pcDNA3.1(+).

### Quantitative PCR

Total RNA was extracted using TRIzol reagent (Invitrogen, Carlsbad, CA, USA) according to the manufacturer’s instructions. After treatment with DNase I (Takara Bio Inc., Shiga, Japan), total RNA (2 μg) was reverse-transcribed to cDNA in a final volume of 20 μL using M-MLV Reverse Transcriptase (Promega, Madison, WI, USA) and oligo-dT18 random primers according to the manufacturer’s instructions. β-actin was used as a standard for gene expression. All primers for the selected genes were designed by Primer Premier 5 (Table 1). SYBR Green real-time PCR Master Mix reagents (Toyobo Co., Ltd., Osaka, Japan), cDNA, ddH_2_O, the sense and antisense primers (200 nmol/L for each gene) were used for quantitative PCR, which was performed using an Mx3005p instrument (Stratagene, La Jolla, CA, USA). The thermal cycling conditions were as follows: 95°C for 1 min, followed by 40 cycles of denaturation at 95°C for 15 s, annealing at different temperature for 15 s, and extension at 72°C for 40 s. Melting curve and sequence analyses were performed for each product to confirm the specific amplification. mRNA expression levels in cells overexpressing DGAT2 are presented as a ratio of those in control cells transfected with pcDNA3.1(+).

**Table 1:** Primers used in RT-PCR analysis

**Gene**	**GenBank access no.**	**Sequence (5’→3’)**	**Product, bp**
HSL	U08188.1	F: AGTGCCTATTCAGGGACAGA	184
		R: TGGGCGATGTGGTCTTTT	
ACACA	AY451393	F: GACAGAGGAAGATGGCGTCC	172
		R: TACAACTTCTGCTCGCTGGG	
a-FABP	NM_024406.2	F: GCTCATAGCACCCTCCTG	93
		R: TCCAGGTTCCCACAAAGG	
LPL	NM_008509.2	F: ACTGCCACTTCAACCACACC	211
		R: GCCACATCATTTCCCACC	
ATGL	NM_025802.3	F: GACCTGATGACCACCCTTTC	169
		R: GGCTACCCGTCTGCTCTTT	
DGAT2	NM_001160080.1	F: GGCTCAATAGGTCCAAGGTA	96
		R: GGGCGTGTTCCAGTCAAA	
SCD1	NM_009127.4	F: GCTCTACACCTGCCTCTTC	103
		R: CCGTGCCTTGTAAGTTCTG	
β-actin	NM_007393.3	F: TAAGGCCAACCGTGAAAAGATGAC	422
		R: ACCGCTCGTTGCCAATAGTGATG	
PPARγ	NM_001127330.1	F: TCAAGGGTGCCAGTTTCGC	232
		R: GGGCTTCCGCAGGCTTTT	
FASN	NM_007988.3	F: CCAAGACTGACTCGGCTACT	280
		R: GCCAGGTTCGGAATGCTAT	
FAT/CD36	NM_001159556.1	F: CTGTGGGCTCATTGCTGG	214
		R: CGCCACGTCATCTGGGTTT	

### Immunoblot analysis

The cells were lysed in RIPA lysis buffer. Homogenates were centrifuged at 12,000 rpm for 5 min at 4°C, and the protein concentration in the supernatants was determined using a BCA protein assay reagent kit (Pierce, Rockford, IL). Protein samples, subjected to a 20% SDS (Beyotime, Shanghai, China), were degenerated for 10 min at 99°C. A total of 30 μg protein were resolved by sodium dodecyl sulfate (SDS)-poly-acrylamide gel electrophoresis (30% acrylamide, 1.5 mol/L Tris (pH8.8), 10% SDS, 10% ammonium persulfate, TEMED; 10% SDS-PAGE) and separated by electrophoresis at 110 V for 75 min using Tris-glycine running buffer (0.025 mol/L Tris base, 0.192 mol/L glycine, and 0.1% SDS, pH 8.3). Proteins then were subsequently electrotransferred onto polyvinylidene difluoride membranes (Millipore, Billerica, MA, USA) using transfer buffer containing 25 mmol/L Tris base, 192 mmol/L glycine, and 10% methanol at pH 8.1-8.3. The membranes were blocked with 5% nonfat milk in PBS for 1 h at room temperature. The primary antibodies [goat anti-LPL, rabbit anti-ATGL, rabbit anti-ACACA, goat anti-SCD1, rabbit anti-CD36 (Santa Cruz Biotechnology Inc., Dallas, Texas, USA); rabbit anti-β-actin (Cell Signaling, Danvers, MA, USA); goat anti-DGAT2, mouse anti-FASN (Lifespan, Providence, RI, USA)] were incubated at 4°C overnight, followed by incubation with the appropriate secondary antibody (1:1,000, Bioss, Beijing, China) for 1 h at room temperature. Protein expression was measured using a FluorChem M Fluorescent Imaging System (ProteinSimple, Santa Clara, CA, USA) and normalized to β-actin expression.

### Statistical analysis

The data are presented as the mean ± SEM. An independent t-test was used for statistical analysis of the differences between the means, and the cut-off point for significance was set at *P* < 0.05.

## Results

### Lipid accumulation in 3T3-L1 adipocytes overexpressing DGAT2

**Figure 1:**
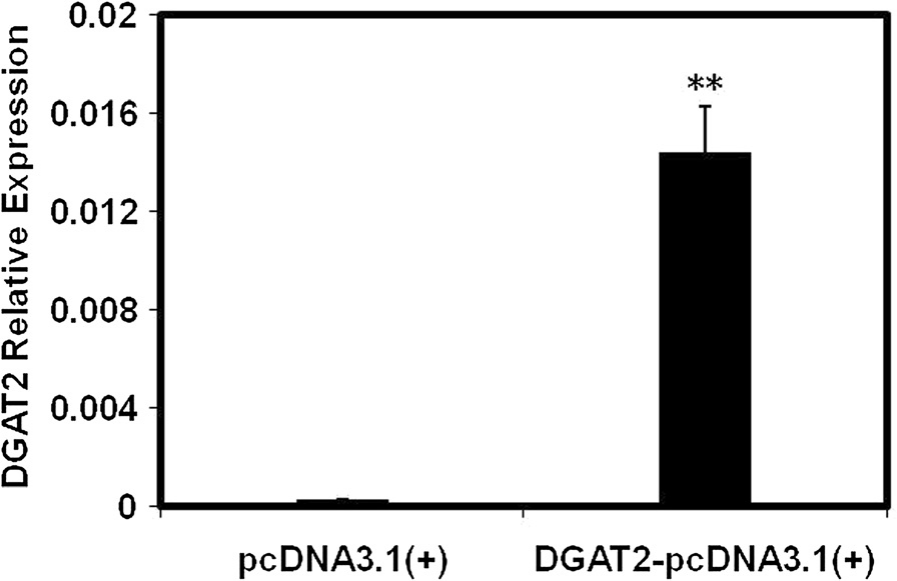
**DGAT2 mRNA expression in 3T3-L1 adipocytes.** DGAT2 mRNA expression was detected by RT-PCR in 3T3-L1 adipocytes after 12 d of differentiation. Cells transfected with the empty pcDNA3.1(+) vectors were used as control cells. Results are presented as the mean ± SEM of six independent cell preparations. **P < 0.01.

**Figure 2:**
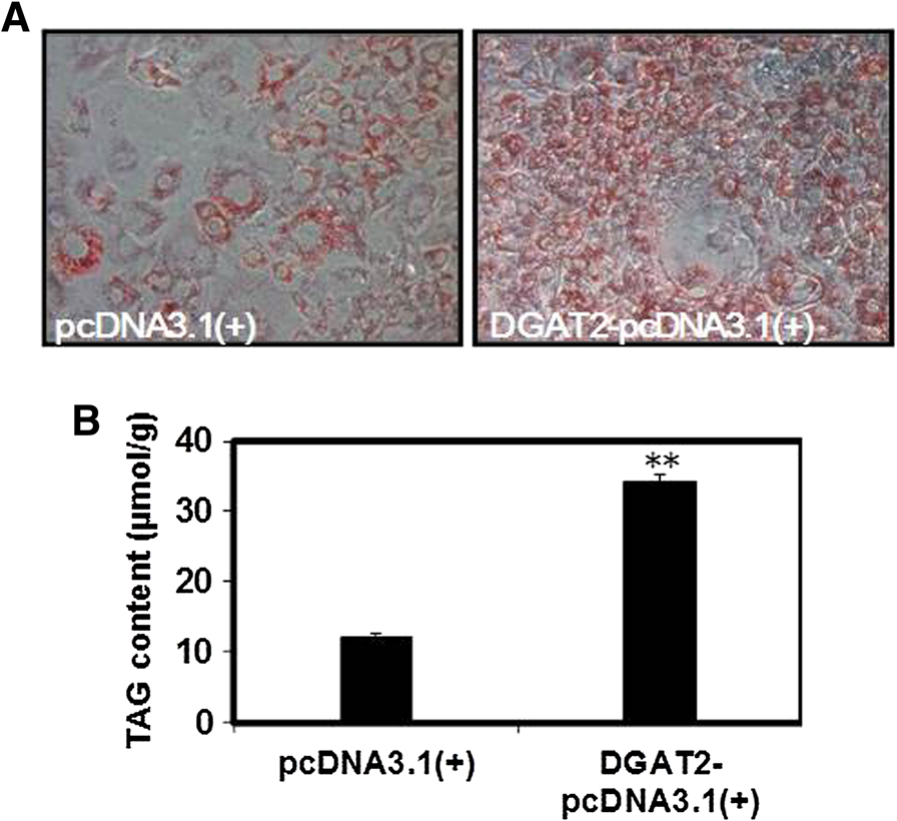
**Effect of DGAT2 overexpression on lipid accumulation.** The cells were incubated with FA-DM (DM containing 10 μmol/L C_16_-palmic acid, C_16_- palmitoleic acid, C_18_-stearic acid, C_18_-oleic acid, C_18_-linoleic acid, and C_20_-arachidic acid bound to 60 μmol/L FA-free BSA) for 12 d of differentiation and collected for Oil Red O staining **(A)** and TAG content determination **(B)**. Results are presented as the mean ± SEM of six independent cell preparations and are shown as μmol/g of cellular protein. **P < 0.01.

3T3-L1 cells transfected with pcDNA3.1(+)-DGAT2 had 63-fold higher DGAT2 mRNA expression than control cells transfected with empty vector (*P* < 0.01) (Figure 1). Oil Red O staining and TAG content analysis similarly demonstrated that lipid accumulation was much higher in 3T3-L1 adipocytes overexpressing DGAT2 (Figure 2).

### FA composition in 3T3-L1 adipocytes overexpressing DGAT2 cultured with DM

After 12 d of induction in DM, the proportion of palmitoleic acid (C16:1) and oleic acid (C18:1) was significantly higher in 3T3-L1 adipocytes overexpressing DGAT2 than in control cells (*P* < 0.05) (Table 2). The proportion of palmitic acid (C16:0) and stearic acid (C18:0) decreased with DGAT2 overexpression, but the difference was not significant (*P* > 0.05) (Table 2).

**Table 2:** FA composition of 3T3-L1 adipocytes overexpressing DGAT2 cultured with DM

**Fatty acids**	**Control**	**DGAT2**	***P *** **value**
Palmitoleic acid (C16:1)	2.17 ± 0.52	4.20 ± 0.27^*^	*P* < 0.05
Palmitic acid (C16:0)	48.03 ± 2.90	41.76 ± 2.10	*P* > 0.05
Linoleic acid (C18:2)	3.17 ± 0.35	2.94 ± 0.13	*P* > 0.05
Oleic acid (C18:1)	18.43 ± 0.13	21.87 ± 0.96^*^	*P* < 0.05
Stearic acid (C18:0)	20.79 ± 1.45	19.97 ± 0.54	*P* > 0.05
Arachidonic acid (C20:4)	7.40 ± 0.75	9.26 ± 0.83	*P* > 0.05

After 12 d of differentiation, cells were collected for FA analysis. Cells were then subjected to transesterification and injected into a GC. The results are expressed as the percent of the total FA content (100%). The data are presented as the mean ± SEM of six independent wells.

*P < 0.05.

### FA composition in 3T3-L1 adipocytes overexpressing DGAT2 cultured with FA-DM

When cells were cultured with FA-DM for 12 d, the proportion of palmitoleic acid (C16:1; *P* < 0.05), oleic acid (C18:1; *P* < 0.01), and linoleic acid (C18:2; *P* < 0.01) was significantly higher in 3T3-L1 adipocytes overexpressing DGAT2 than in control cells (Table 3). The reverse was true for stearic acid (C18:0) and arachidonic acid (C20:4) (*P* < 0.01; Table 3). When the cells were incubated in FA-DM for 30 min, the proportion of palmitoleic acid (C16:1) was slightly elevated in cells overexpressing DGAT2 (*P* < 0.05) (Table 3).

**Table 3:** FA composition of 3T3-L1 adipocytes overexpressing DGAT2 cultured with FA-DM

**Fatty acids**	**30 min**		**12 d**	
	**Control**	**DGAT2**	**Control**	**DGAT2**
Myristic acid (C14:0)	2.70 ± 0.15	2.62 ± 0.49	2.84 ± 0.42	2.42 ± 0.29
Palmitoleic acid (C16:1)	2.67 ± 0.15	3.86 ± 0.33^*^	2.61 ± 0.17	3.54 ± 0.33^*^
Palmitic acid (C16:0)	39.11 ± 4.55	33.61 ± 2.104	31.17 ± 1.1003	29.23 ± 1.34
Linoleic acid (C18:2)	5.14 ± 0.38	6.63 ± 1.74	8.44 ± 0.29	11.38 ± 0.41^**^
Oleic acid (C18:1)	14.18 ± 1.19	12.31 ± 1.07	8.66 ± 0.32	16.47 ± 0.52^**^
Stearic acid (C18:0)	22.95 ± 2.33	26.45 ± 0.99	26.76 ± 0.42	22.54 ± 0.90^**^
Arachidonic acid (C20:4)	12.29 ± 1.88	13.56 ± 1.99	15.65 ± 0.46	9.86 ± 1.10^**^
Arachidic acid (C20:0)	0.96 ± 0.04	0.96 ± 0.15	4.73 ± 0.21	4.55 ± 0.36

3T3-L1 adipocytes were incubated with FA-DM (10 μmol/L C_16_-palmic acid, C_16_-palmitoleic acid, C_18_-stearic acid, C_18_-oleic acid, C_18_-linoleic acid, and C_20_-arachidic acid bound to 60 μmol/L FA-free BSA) for 30 min or 12 d. Cells were then subjected to transesterification and injected into a GC for FA analysis. The results are expressed as the percent of the total FA content (100%). The data are presented as the mean ± SEM of six independent wells.

*P < 0.05; **P < 0.01.

### FA composition in 3T3-L1 adipocytes overexpressing DGAT2 cultured with IFA-DM

**Figure 3:**
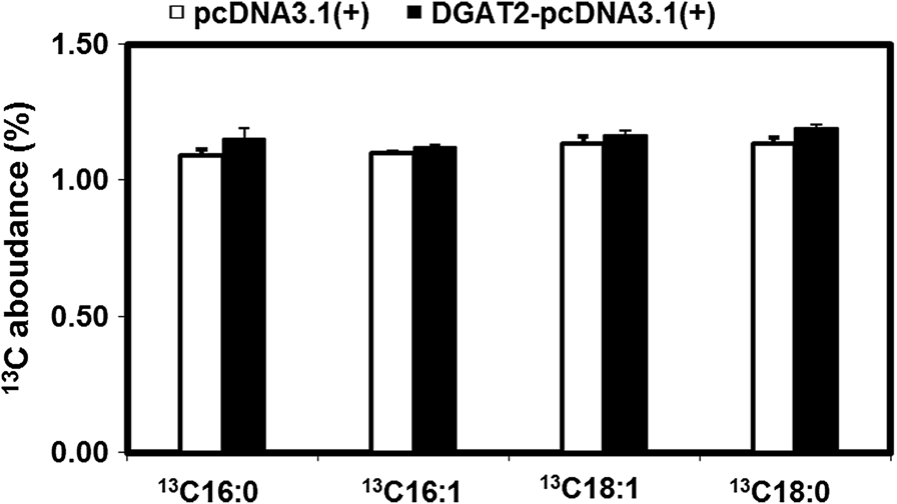
**Effect of DGAT2 overexpression on **^**13**^**C-labeled FA accumulation in adipocytes.** The cells were incubated with IFA-DM (DM containing 10 μmol/L ^13^C_16_-palmic acid, C_16_-palmitoleic acid, ^13^C_18_-stearic acid, ^13^C_18_-oleic acid, C_18_-linoleic acid, and C_20_-arachidic acid bound to 60 μmol/L FA-free BSA). After 12 d of differentiation, the mature adipocytes were collected for FA analysis by GC-IRMS. The results are expressed as the percentage of each FA (%). Data are presented as the mean ± SEM of six independent wells.

When cells were cultured with IFA-DM for 12 d, the proportion of ^13^C FAs were the same in 3T3-L1 adipocytes overexpressing DGAT2 and control cells (*P* > 0.05) (Figure 3). These results indicate that the higher proportion of C16:1 and C18:1 may originate from *de novo* FA synthesis but not from the uptake of specific FAs from the medium.

### Adipogenic gene expression in 3T3-L1 adipocytes overexpressing DGAT2

**Figure 4:**
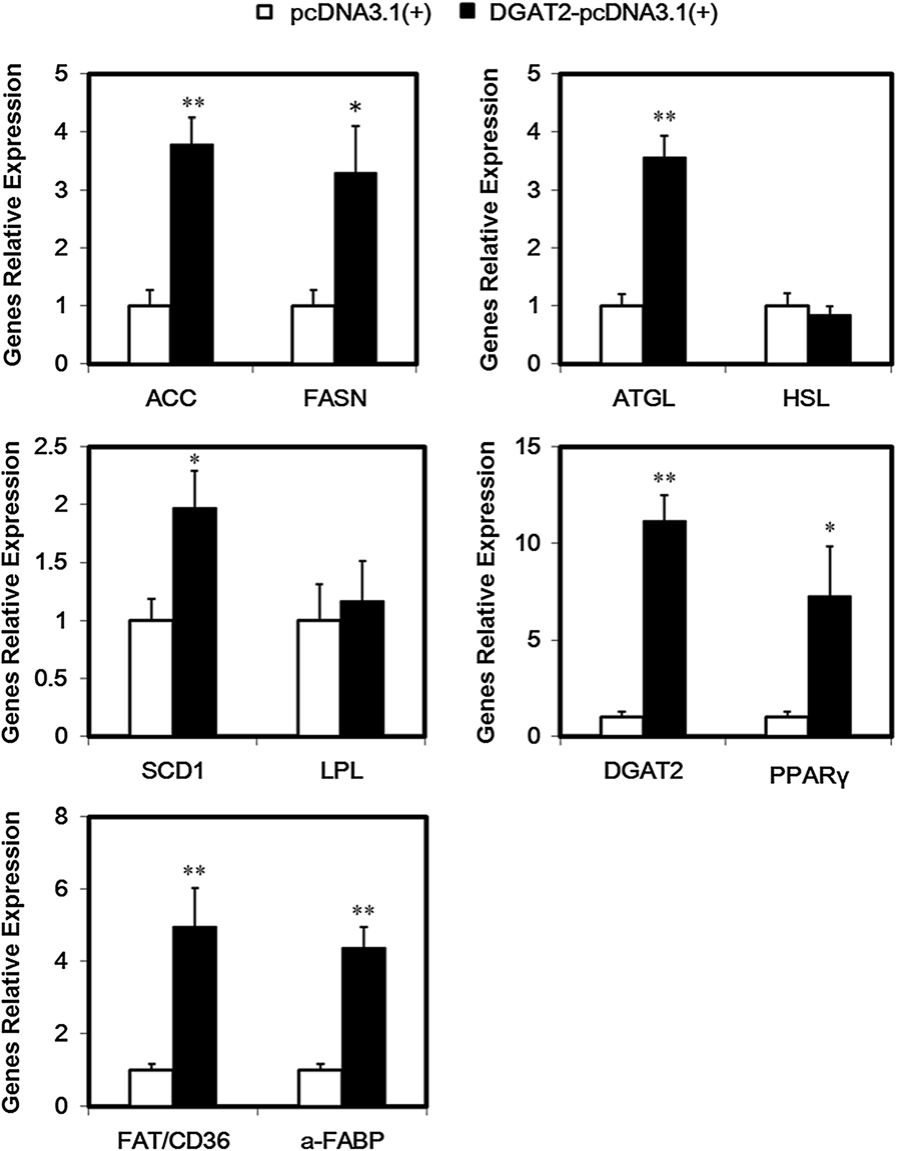
**Effect of DGAT2 overexpression on adipogenic gene mRNA expression.** The cells were incubated with FA-DM (DM containing 10 μmol/L C_16_-palmic acid, C_16_-palmitoleic acid, C_18_-stearic acid, C_18_-oleic acid, C_18_-linoleic acid, and C_20_-arachidic acid bound to 60 μmol/L FA-free BSA). After 12 d of differentiation, total RNA in mature adipocytes were isolated for quantitative PCR. Adipocytes transfected with the empty pcDNA3.1(+) vectors were control cells. Results are presented as the mean ± SEM of six independent cell preparations. *P < 0.05, **P < 0.01.

**Figure 5:**
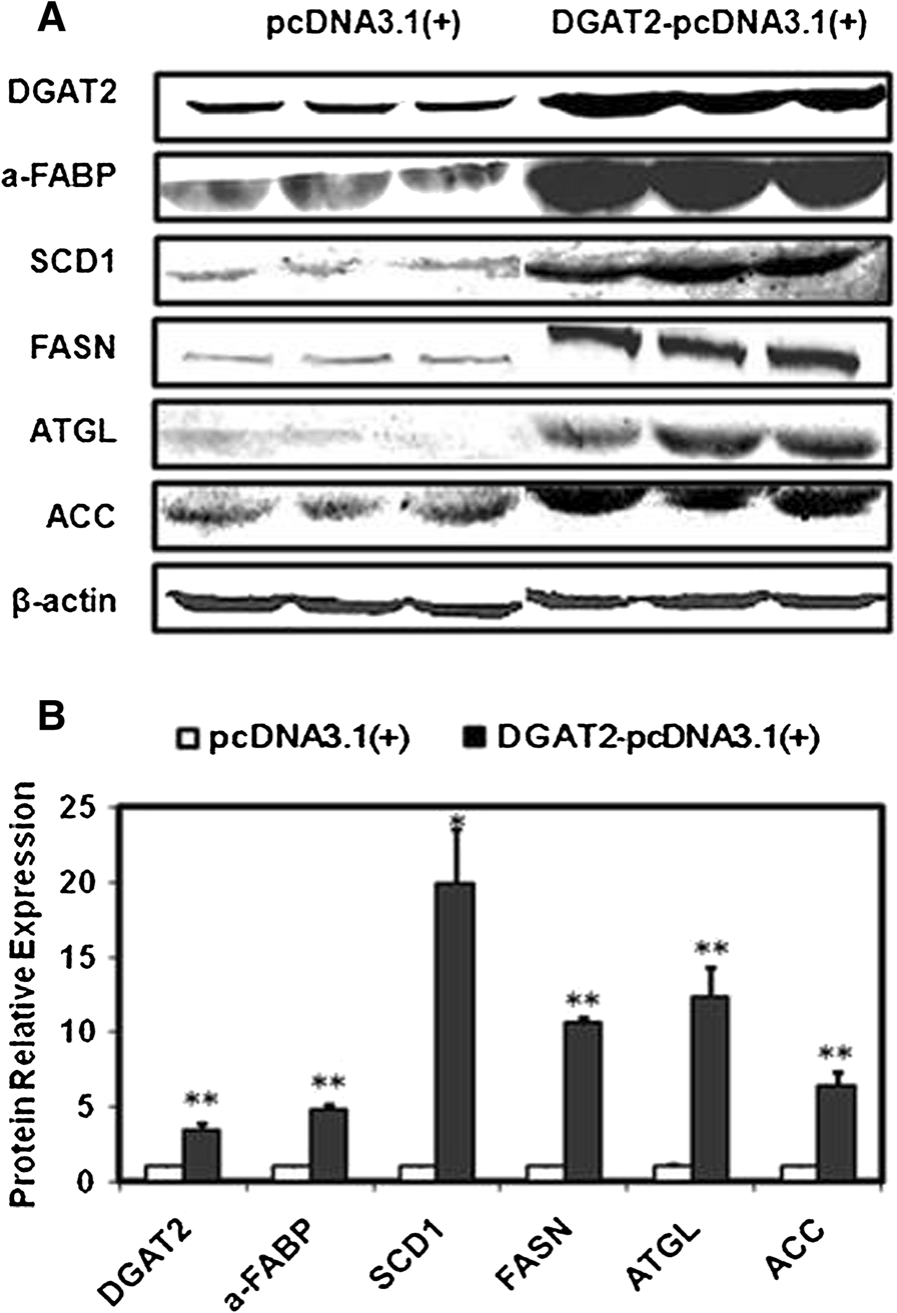
**Effect of DGAT2 overexpression on adipogenic gene protein expression.** The cells were incubated with FA-DM (DM containing 10 μmol/L. C_16_-palmic acid, C_16_-palmitoleic acid, C_18_-stearic acid, C_18_-oleic acid, C_18_-linoleic acid, and C_20_-arachidic acid bound to 60 μmol/L FA-free BSA). After 12 d of differentiation, total protein in mature adipocytes was extracted for western blotting. **(A)** Western blotting images; **(B)** Statistical analysis of protein expression using Image J software. Cells transfected with the empty pcDNA3.1(+) vector were control cells. Results are presented as the mean ± SEM of three independent cell preparations. *P < 0.05, **P < 0.01.

As shown in Figures 4 and 5, mRNA and protein expression of adipose triglyceride lipase, acetyl CoA carboxylase (ACACA), FA synthase (FASN), stearoyl-CoA desaturase-1 (SCD1), and FA-binding protein (a-FABP) was significantly higher in 3T3-L1 adipocytes overexpressing DGAT2 than in control cells. In addition, mRNA expression of FA translocase (FAT/CD36) and peroxisomal proliferator-activated receptor γ (PPARγ) was higher in cells overexpressing DGAT2.

## Discussion

Previous studies have proved that DGAT2 plays an important role in lipid accumulation [17]. However, little is known about the influence of DGAT2 on the FA composition of adipocytes. Our study revealed that TAG accumulation, the proportion of MUFAs, and the expression of adipogenic genes were all higher in 3T3-L1 cells overexpressing DGAT2 than in control cells.

The existence of DGAT2 was first implicated from the finding that mice lacking DGAT1 had an abundance of TAG in their tissues [22]. Further research revealed that DGAT2 mRNA was highly expressed in several lipid metabolism tissues, including liver [23], mammary glands [24], and adipose tissue [25]. DGAT2 overexpression enhanced the accumulation of lipid droplets in COS-7 cells [17]. In contrast, DGAT2 knockdown decreased lipid storage in 3T3-L1 adipocytes [18]. Consistent with these studies, our results showed that the TAG content was significantly higher in 3T3-L1 adipocytes overexpressing DGAT2 than in control cells.

TAG formation is catalyzed by both DGAT1 and DGAT2, through the acylation of diacylglycerol [14,15]. In mice, DGAT1 is known to have a strong substrate preference for incorporating oleoyl-CoA into TAG [15,16]. Gene polymorphisms of DGAT in cows were reported to be closely related to the content of oleic acid in muscle [26]. Our results also demonstrated the novel function of porcine DGAT2 on the FA composition of adipocytes. When cultured in either DM or FA-DM for 12 d, 3T3-L1 adipocytes overexpressing DGAT2 showed a higher proportion of MUFAs (C16:1 and C18:1); when incubated in FA-DM for 30 min, the proportion of only palmitoleic acid (C16:1) was slightly elevated in cells overexpressing DGAT2 than in controls. However, when the cells were incubated with IFA-DM containing a stable IFA mixture, the proportion of ^13^C FAs was unchanged. These observations indicate that the long-term effects of DGAT2 overexpression in the FA profile may originate from *de novo* FA synthesis but not from the uptake of specific FAs from the culture medium.

To investigate the possible mechanism underlying the effects of DGAT2 on FA composition, we studied the expression of adipogenic genes in cells overexpressing DGAT2 mRNA and protein expression of ACACA, FASN, A-FABP, and SCD1 was significantly higher in 3T3-L1 adipocytes overexpressing DGAT2 than in control cells, as was the mRNA expression of FAT/CD36 and PPARγ. Of these genes, ACACA and FASN are the two key enzymes in *de novo* FA synthesis. This evidence suggests that the higher levels of lipid accumulation observed in cells overexpressing DGAT2 may result from *de novo* FA synthesis. Several studies have demonstrated that FAT/CD36, a-FABP, and SCD1 are associated with the uptake of MUFAs [11,13,27]. DGAT2 and SCD1 co-localize to a compartment involved in activating lipid synthesis, suggesting that SCD1 and DGAT2 play a coordinated role in TAG synthesis [28]. Thus, SCD1 may participate in TAG synthesis by producing an easily accessible pool of MUFA [29].

Although the function of DGAT2 has been described solely as the catalysis of TAG formation, we found that the expression of a great number of adipogenic genes was affected by DGAT2 overexpression. DAG, an important cellular second messenger, may be involved in this phenomenon. Considering that DGAT catalyzes TAG synthesis through DAG, numerous reports have shown that lower DGAT2 expression resulted in lower DAG content [30,31], subsequently activating protein kinase Cϵ (PKCϵ) [32-35]. Therefore, we suspect that the expression of various adipogenic genes may be modulated by PKCϵ, which could increase the transcription of genes involved in FA biosynthesis by activating the sterol regulatory element binding protein 1c(SREBP1) [36-39]. The elucidation of the precise mechanism whereby DGAT2 affects adipogenic gene expression will require further study.

In conclusion, our study revealed that TAG accumulation, the cellular proportion of MUFAs, and the expression of adipogenic genes were higher in 3T3-L1 adipocytes overexpressing DGAT2 than in control cells. This information may be helpful in producing and selecting animals with a desirable FA profile.

## Abbreviations

FA: Fatty acid; DGAT2: Diacylglycerol acyltransferase 2; DM: Differentiation medium; FA-DM: Differentiation medium with a mixture of FA; IFA-DM: Differentiation medium with ^13^C stable isotope-labeled FA mixture; MUFAs: Monounsaturated fatty acids; TAG: Triacylglyceride; GC: Gas chromatography; FBS: Fetal bovine serum; BSA: Bovine serum albumin; IR-MS: Isotope ratio mass spectrometry; ACACA: Acetyl CoA carboxylase; FASN: FA synthase; SCD1: Stearoyl-CoA desaturase-1; a-FABP: FA-binding protein.

## Competing interests

The authors declare that they have no competing interests.

## Authors’ contributions

GJM participated in the design of the study, carried out the experiments and statistical analysis, and wrote the first draft of the manuscript. SG and JQY participated in the design of the study and the statistical analysis, and oversaw manuscript preparation. ZXT and CH participated in the cell experiments and plasmid construction. WSB, WLN, GP, XQY, ZYL, and YL participated in the study design and coordination. ZZQ participated in writing the final versions of the manuscript. All authors have read and approved the final manuscript.

## References

[B1] Cameron ND Enser M Nute GR Nute GR Whittington FM Penman JC Fiske AC Perry AM Wood JD Genotype with nutrition interaction on fatty acid composition of intramuscular fat and the relationship with flavour of pig meat Meat Sci 2000 55 2 187 195 10.1016/S0309-1740(99)00142-4 22061084

[B2] Keys A Anderson JT Grande F Serum cholesterol response to changes in the diet: IV. Particular saturated fatty acids in the diet Metabolism 1965 14 776 787 10.1016/0026-0495(65)90004-1 25286466

[B3] Sirtori CR Tremoli E Gatti E Montanari G Sirtori M Colli S Gianfranceschi G Maderna P Dentone CZ Testolin G Controlled evaluation of fat intake in the Mediterranean diet: comparative activities of olive oil and corn oil on plasma lipids and platelets in high-risk patients Am J Clin Nutr 1986 44 635 642 3094360 10.1093/ajcn/44.5.635

[B4] McDonald BE Gerrard JM Bruce VM Corner EJ Comparison of the effect of canola oil and sunflower oil on plasma lipids and lipoproteins and on in vivo thromboxane A2 and prostacyclin production in healthy young men Am J Clin Nutr 1989 50 1382 1388 2596428 10.1093/ajcn/50.6.1382

[B5] Lopez-Segura F Velasco F Lopez-Miranda J Castro P Lopez-Pedrera R Blanco A Jimenez-Pereperez J Torres A Trujillo J Ordovas J Monounsaturated fatty acid–enriched diet decreases plasma plasminogen activator inhibitor type 1 Arterioscl Throm Vas 1996 16 82 88 10.1161/01.ATV.16.1.82 8548431

[B6] Coleman RA Lee DP Enzymes of triacylglycerol synthesis and their regulation Prog Lipid Res 2004 43 134 176 10.1016/S0163-7827(03)00051-1 14654091

[B7] Hollenback D Bonham L Law L Rossnagle E Romero L Carew H Tompkins CK Leung DW Singer JW White T Substrate specificity of lysophosphatidic acid acyltransferase beta - evidence from membrane and whole cell assays J Lipid Res 2006 47 593 604 16369050 10.1194/jlr.M500435-JLR200

[B8] Sprecher H Metabolism of highly unsaturated n-3 and n-6 fatty acids BBA-Mol cell Biol L 2000 1486 219 231 10.1016/s1388-1981(00)00077-9 10903473

[B9] Abumrad N Coburn C Ibrahimi A Membrane proteins implicated in long-chain fatty acid uptake by mammalian cells: CD36, FATP and FABPm BBA-Mol Cell Blol L 1999 1441 4 13 10.1016/s1388-1981(99)00137-7 10526223

[B10] Abumrad N Harmon C Ibrahimi A Membrane transport of long-chain fatty acids: evidence for a facilitated process J Lipid Res 1998 39 2309 2318 9831619

[B11] Ibrahimi A Sfeir Z Magharaie H Amri EZ Grimaldi P Abumrad NA Expression of the CD36 homolog (FAT) in fibroblast cells: effects on fatty acid transport Proc Natl Acad Sci U S A 1996 93 2646 2651 10.1073/pnas.93.7.2646 8610095 PMC39684

[B12] Laugerette F Passilly-Degrace P Patris B Niot I Febbraio M Montmayeur JP Besnard P CD36 involvement in orosensory detection of dietary lipids, spontaneous fat preference, and digestive secretions J Clin Invest 2005 115 3177 3184 10.1172/JCI25299 16276419 PMC1265871

[B13] Schaffer JE Lodish HF Expression cloning and characterization of a novel adipocyte long chain fatty acid transport protein Cell 1994 79 427 436 10.1016/0092-8674(94)90252-6 7954810

[B14] Cases S Smith SJ Zheng YW Myers HM Lear SR Sande E Novak S Collins C Welch CB Lusis AJ Identification of a gene encoding an acyl CoA: diacylglycerol acyltransferase, a key enzyme in triacylglycerol synthesis Proc Natl Acad Sci U S A 1998 95 13018 13023 10.1073/pnas.95.22.13018 9789033 PMC23692

[B15] Cases S Stone SJ Zhou P Yen E Tow B Lardizabal KD Voelker T Farese RV Jr Cloning of DGAT2, a second mammalian diacylglycerol acyltransferase, and related family members J Biol Chem 2001 276 38870 38876 10.1074/jbc.M106219200 11481335

[B16] Chen HC Smith SJ Ladha Z Jensen DR Ferreira LD Pulawa LK McGuire JG Pitas RE Eckel RH Farese RV Jr Increased insulin and leptin sensitivity in mice lacking acyl CoA: diacylglycerol acyltransferase 1 J Clin Invest 2002 109 1049 1056 10.1172/JCI0214672 11956242 PMC150948

[B17] McFie PJ Banman SL Kary S Stone SJ Murine diacylglycerol acyltransferase-2 (DGAT2) can catalyze triacylglycerol synthesis and promote lipid droplet formation independent of its localization to the endoplasmic reticulum J Biol Chem 2011 286 28235 28246 10.1074/jbc.M111.256008 21680734 PMC3151068

[B18] Nian Z Sun Z Yu L Toh SY Sang J Li P Fat-specific protein 27 undergoes ubiquitin-dependent degradation regulated by triacylglycerol synthesis and lipid droplet formation J Biol Chem 2010 285 9604 9615 10.1074/jbc.M109.043786 20089860 PMC2843210

[B19] Student A Hsu R Lane M Induction of fatty acid synthetase synthesis in differentiating 3T3-L1 preadipocytes J Biol Chem 1980 255 4745 4750 7372608

[B20] Folch J Lees M Sloane-Stanley G A simple method for the isolation and purification of total lipids from animal tissues J Biol Chem 1957 226 497 509 13428781

[B21] Morrison WR Smith LM Preparation of fatty acid methyl esters and dimethylacetals from lipids with boron fluoride–methanol J Lipid Res 1964 5 600 608 14221106

[B22] Smith SJ Cases S Jensen DR Chen HC Sande E Tow B Sanan DA Raber J Eckel RH Farese RV Obesity resistance and multiple mechanisms of triglyceride synthesis in mice lacking Dgat Nat Genet 2000 25 87 90 10.1038/75651 10802663

[B23] Yu YH Ginsberg N The role of acyl-CoA: diacylglycerol acyltransferase (DGAT) in energy metabolism Ann Med 2004 36 252 261 10.1080/07853890410028429 15224651

[B24] Johnson AC Stahl A Zager RA Triglyceride accumulation in injured renal tubular cells: alterations in both synthetic and catabolic pathways Kidney Int 2005 67 2196 2209 10.1111/j.1523-1755.2005.00325.x 15882263

[B25] Suzuki R Tobe K Aoyama M Sakamoto K Ohsugi M Kamei N Nemoto S Inoue A Ito Y Uchida S Hara K Yamauchi T Kubota N Erauchi Y Adowaki T Expression of DGAT2 in white adipose tissue is regulated by central leptin action J Biol Chem 2005 280 3331 3337 10.1074/jbc.M410955200 15550388

[B26] Chris Kazala E Lozeman FJ Mir PS Fatty acid composition of muscle fat and enzymes of storage lipid synthesis in whole muscle from beef cattle Lipids 2006 41 11 1049 1057 10.1007/s11745-006-5055-0 17263304

[B27] Sha R Kane C Xu Z Banaszak L Ernlohr D Modulation of ligand binding affinity of the adipocyte lipid-binding protein by selective mutation J Biol Chem 1993 268 7885 7892 8463312

[B28] Rusinol AE Cui Z Chen MH Vance JE A unique mitochondria- associated membrane fraction from rat liver has a high capacity for lipid synthesis and contains pre-Golgi secretory proteins including nascent lipoproteins J Biol Chem 1994 269 27494 27502 7961664

[B29] Man WC Miyazaki M Chu K Ntambi J Colocalization of SCD1 and DGAT2: implying preference for endogenous monounsaturated fatty acids in triglyceride synthesis J Clin Invest 2006 47 1928 1939 10.1194/jlr.M600172-JLR200 16751624

[B30] Jornayvaz FR Jurczak MJ Samuel VT Shulman GI Hepatic steatosis and diacylglycerol-mediated hepatic insulin resistance in acyl-CoA: diacylglycerol acyltransferase 2 (DGAT2) transgenic mice Proc Natl Acad Sci U S A 2011 108 E524 E524 10.1073/pnas.1109195108 PMC3078388 21436037

[B31] Monetti M Levin MC Watt MJ Sajan MP Marmor S Hubbard BK Stevens RD Bain JR Newgard CB Farese RV Dissociation of hepatic steatosis and insulin resistance in mice overexpressing DGAT in the liver Cell Metab 2007 6 69 78 10.1016/j.cmet.2007.05.005 17618857

[B32] Considine RV Nyce MR Allen LE Morales LM Triester S Serrano J Colberg J Lanza-Jacoby S Caro JF Protein kinase C is increased in the liver of humans and rats with non-insulin-dependent diabetes mellitus: an alteration not due to hyperglycemia J Clin Invest 1995 95 2938 2944 10.1172/JCI118001 7769136 PMC295982

[B33] Liu L Zhang Y Chen N Shi X Tsang B Yu Y Upregulation of myocellular DGAT1 augments triglyceride synthesis in skeletal muscle and protects against fat-induced insulin resistance J Clin Invest 2007 117 1679 1689 10.1172/JCI30565 17510710 PMC1866250

[B34] Samuel VT Liu Z Wang A Beddow SA Geisler JG Kahn M Zhang X Monia BP Bhanot S Shulman GI Inhibition of protein kinase Cepsilon prevents hepatic insulin resistance in nonalcoholic fatty liver disease J Clin Invest 2007 117 739 745 10.1172/JCI30400 17318260 PMC1797607

[B35] Yu C Chen YJ Mechanism by which fatty acids inhibit insulin activation of insulin receptor substrate-1 (IRS-1)-associated phosphatidylinositol 3-kinase activity in muscle J Biol Chem 2002 277 50230 50236 10.1074/jbc.M200958200 12006582

[B36] Choi CS Savage DB Kulkarni A Yu XX Liu ZX Morino K Kim S Distefano A Samuel VT Neschen S Suppression of diacylglycerol acyltransferase-2 (DGAT2), but not DGAT1, with antisense oligonucleotides reverses diet-induced hepatic steatosis and insulin resistance J Biol Chem 2007 282 22678 22688 10.1074/jbc.M704213200 17526931

[B37] Farese RV Sajan MP Standaert ML Insulin-sensitive protein kinases (atypical protein kinase C and protein kinase B/Akt): actions and defects in obesity and type II diabetes Exp Biol Med 2005 230 593 605 10.1177/153537020523000901 16179727

[B38] Matsumoto M Ogawa W Akimoto K Inoue H Miyake K Furukawa K Hayashi Y Iguchi H Matsuki Y Hiramatsu R PKClambda in liver mediates insulin-induced SREBP-1c expression and determines both hepatic lipid content and overall insulin sensitivity J Clin Invest 2003 112 935 944 10.1172/JCI200318816 12975478 PMC193669

[B39] Shimomura I Matsuda M Hammer RE Bashmakov Y Brown MS Goldstein JL Decreased IRS-2 and increased SREBP-1c lead to mixed insulin resistance and sensitivity in livers of lipodystrophic and ob/ob mice Mol Cell Biol 2000 6 77 86 10949029

